# DeepBTS: Prediction of Recurrence-free Survival of Non-small Cell Lung Cancer Using a Time-binned Deep Neural Network

**DOI:** 10.1038/s41598-020-58722-z

**Published:** 2020-02-06

**Authors:** Bora Lee, Sang Hoon Chun, Ji Hyung Hong, In Sook Woo, Seoree Kim, Joon Won Jeong, Jae Jun Kim, Hyun Woo Lee, Sae Jung Na, Kyongmin Sarah Beck, Bomi Gil, Sungsoo Park, Ho Jung An, Yoon Ho Ko

**Affiliations:** 1 Deargen Inc., Daejeon, Korea; 2 Division of Oncology, Department of Internal Medicine, College of Medicine, The Catholic University of Korea, Seoul, Republic of Korea; 3 Department of Thoracic and Cardiovascular Surgery, College of Medicine, The Catholic University of Korea, Seoul, Korea; 4 Department of Hematology-Oncology, Ajou University School of Medicine, Suwon, Republic of Korea; 5 Department of Radiology, College of Medicine, The Catholic University of Korea, Seoul, Republic of Korea; 6 Cancer Research Institute, College of Medicine, The Catholic University of Korea, Seoul, Republic of Korea

**Keywords:** Non-small-cell lung cancer, Computational science

## Abstract

Accurate prediction of non-small cell lung cancer (NSCLC) prognosis after surgery remains challenging. The Cox proportional hazard (PH) model is widely used, however, there are some limitations associated with it. In this study, we developed novel neural network models called binned time survival analysis (DeepBTS) models using 30 clinico-pathological features of surgically resected NSCLC patients (training cohort, *n* = 1,022; external validation cohort, *n* = 298). We employed the root-mean-square error (in the supervised learning model, s- DeepBTS) or negative log-likelihood (in the semi-unsupervised learning model, su-DeepBTS) as the loss function. The su-DeepBTS algorithm achieved better performance (C-index = 0.7306; AUC = 0.7677) than the other models (Cox PH: C-index = 0.7048 and AUC = 0.7390; s-DeepBTS: C-index = 0.7126 and AUC = 0.7420). The top 14 features were selected using su-DeepBTS model as a selector and could distinguish the low- and high-risk groups in the training cohort (*p* = 1.86 × 10^−11^) and validation cohort (*p* = 1.04 × 10^−10^). When trained with the optimal feature set for each model, the su-DeepBTS model could predict the prognoses of NSCLC better than the traditional model, especially in stage I patients. Follow-up studies using combined radiological, pathological imaging, and genomic data to enhance the performance of our model are ongoing.

## Introduction

Lung cancer is the fourth most commonly diagnosed cancer and the second most common cause of cancer-related death worldwide. Despite advances in cancer treatment over the last decade, the 5-year survival rate is still around 50% for surgically resected non-small cell lung cancer (NSCLC). Even for stage I patients, 20% showed recurrence within 5 years. Thus, the identification of patients with poor prognoses after surgery is of considerable clinical relevance.

The Cox proportional hazards (PH) model is traditionally used to predict the clinical outcomes or hazard functions corresponding to specific time units. However, this model has the following major drawbacks:

(1) The proportional hazard assumption and linearity of each variable must be satisfied. These assumptions are difficult to be satisfied using real-world data, and their violation may lead to the creation of a false model^1^, (2) the exact model formula for tied samples is not computationally efficient; therefore, Efron’s or Breslow’s approximations are employed to fit the model in a reasonable time. These approximations are incapable of handling ties correctly and produce significantly different results depending on the frequency of ties^2^.

To solve these problems in classical survival analysis, several neural network-based hazard functions and overall survival time prediction models have been developed^3^. Numerous authors have proposed discrete-time deep learning models to predict the risk probability in each time interval^4–7^. These models are efficient, however, the output that represents the risk probability of each time interval must be clearly pre-defined before training. In addition, as the deep-learning models are considered to be ‘black-boxes,’ they provide little insight on which variables have the highest influence on the model^8^. Thus, in this paper, we present a novel neural network model using clinico-pathological variables for predicting the recurrence probabilities of NSCLC patients in time-series intervals after surgical resection. Because few assumptions are needed in the proposed model, it can effectively address the main disadvantages of the Cox PH model and also minimize the effort of producing output data in current neural network model. A novel feature selection method using the neural network model is also proposed, which can be used to measure the effect of each variable on the model.

## Results

### Patient characteristics

The clinico-pathological characteristics for the training and external validation cohorts are summarized in Table 1. In the training cohort, the median age was 65 (33–86) years, 65.2% were male, and 54.3% were former or current smokers. Two-thirds of the patients (63.7%) exhibited adenocarcinoma histology, and 65.7% were classified into stage I. More than half of the tumors (60.4%) were moderately differentiated, with a median tumor size of 2.5 cm. The baseline characteristics in the external validation cohort were not significantly different from those in the training cohort. The median follow-up periods for the training and external validation cohorts were 40.4 and 39.8 months, respectively.

**Table 1:** Baseline characteristics of the training and validation cohorts.

Characteristic		Training cohort	External validation cohort	*p*-value
		*n* = 1,022 (%)	*n* = 298 (%)	
Age (years)	Median (range)	66 (33–86)	66 (25–85)	0.387
Gender	Male	666 (65.2)	195 (65.4)	0.931
	Female	356 (34.8)	103 (34.6)	
Smoking history	Never	461 (45.7)	132 (45.2)	0.884
	Former/current	548 (54.3)	160 (54.8)	
ECOG	0	503 (49.2)	157 (52.7)	0.292
performance status	1	519 (50.8)	141 (47.3)	
CEA	ng/mL	2.1 (1.0–230.1)	1.9 (1.0–1070.9)	0.428
WBC	10^6^/L	7,399 ± 3.8	7,611 ± 3.3	0.381
Neutrophil	%	59.7 ± 31.1	60.0 ± 12.4	0.722
lymphocyte	%	29.9 ± 18.7	28.5 ± 10.9	0.227
Haemoglobin	g/dL	13.2 ± 3.8	13.0 ± 1.7	0.518
Platelet	10^9^/L	237 ± 79	239 ± 83	0.610
C-reactive protein	mg/dL	0.14 (0.0–34.1)	0.12 (0.02–23.6)	0.450
Pulmonary function	FEV1 (L)	2.4 (0.08–352.0)	2.37 (0.96–139.0)	0.915
	DLCo (%)	85 (8–173)	83 (9–159)	0.327
Histology	Adenocarcinoma	651 (63.7)	2003 (67.1)	0.442
	Squamous	303 (29.6)	77 (25.8)	
	others	68 (6.7)	21 (7.0)	
Tumour size	cm	2.5 (0.4–13.0)	2.5 (0.3–13.0)	0.226
No. of LN positivity		0 (0–23)	0 (0-31)	0.847
T stage	T1	474 (46.5)	160 (53.7)	0.064
	T2	433 (42.5)	114 (38.3)	
	T3/4	113 (11.1)	24 (8.1)	
N stage	N0	757 (74.9)	239 (80.5)	0.136
	N1	135 (13.4)	30 (10.1)	
	N2	119 (11.8)	28 (9.4)	
TNM stage	I	669 (65.7)	211 (71.0)	0.218
	II	200 (19.6)	50 (16.8)	
	III	150 (14.7)	36 (12.1)	
Tumor differentiation	Well	197 (19.7)	75 (25.6)	0.054
	Moderately	603 (60.4)	156 (53.2)	
	Poorly	199 (19.9)	62 (21.1)	
Vascular invasion	Yes	143 (14.0)	35 (11.9)	0.489
Lymphatic invasion	Yes	353 (34.6)	95 (32.0)	0.560
Perineural invasion	Yes	59 (5.8)	13 (4.4)	0.642
Resection status*	R0	980 (97.5)	289 (98.0)	0.849
	R1	19 (1.9)	5 (1.7)	
	R2	6 (0.6)	1 (0.3)	
Neoadjuvant treatment	Yes	50 (4.9)	14 (4.7)	0.888
Adjuvant treatment	Yes	333 (33.1)	86 (29.3)	0.214
Recurrence	Yes	272 (26.6)	76 (25.2)	0.618

ECOG, Eastern Cooperative Oncology Group; CEA, carcinoembryonic antigen; WBC, white blood cell; FEV1, forced expiratory volume in the first second; DLCo, diffusing capacity of the lung for carbon monoxide; LN, lymph node.

*R0, number of cancer cells seen microscopically at the resection margin; R1, microscopic positive margin; R2, macroscopic positive margin.

### Model performance

In this study, two discrete-time deep learning models (supervised binned-time survival analysis [s- DeepBTS] and semi-unsupervised binned-time survival analysis [su-DeepBTS]) were compared with the Cox PH model. The performance scores of the models are shown in Table 2. In the training cohort, the proposed su-DeepBTS algorithm performed the best among the three models: a concordance index (C-index) of 0.7306 and an area under the curve (AUC) of 0.7677 were observed for the su-DeepBTS algorithm, while C-index of 0.7048 and 0.7126 and AUCs of 0.7390 and 0.7420 were observed for the Cox PH model and s-DeepBTS algorithm, respectively. The result of one-way ANOVA with *post hoc* test (pairwise *t* test with Holm-Sidak correction) showed the significant difference of C-index between Cox PH model and su-DeepBTS model (*p*-value = 5.45 ×10^-6^). The detailed scores for all of the iterations are provided in Supplementary Table S1.

**Table 2:** Performance scores of three different models.

Number of features		Training cohort		External validation cohort	
		28	Optimal feature set	28	Optimal feature set
Cox PH	C-index	0.7048 ± 0.0067	0.7248 ± 0.0030	0.6939 ± 0.0017	0.6924 ± 0.0009
	AUC	0.7390 ± 0.0071	0.7622 ± 0.0041	0.7064 ± 0.0016	0.7112 ± 0.0010
s-DeepBTS	C-index	0.7126 ± 0.0089	0.7338 ± 0.0022	0.6879 ± 0.0048	0.6944 ± 0.0008
	AUC	0.7420 ± 0.0183	0.7727 ± 0.0024	0.7020 ± 0.0054	0.7083 ± 0.0012
su-DeepBTS	C-index	0.7306 ± 0.0042	0.7419 ± 0.0044	0.7077 ± 0.0019	0.7013 ± 0.0018
	AUC	0.7677 ± 0.0049	0.7780 ± 0.0054	0.7224 ± 0.0021	0.7123 ± 0.0021

Cox PH, Cox proportional-hazards; AUC, area under the curve; s-DeepBTS, supervised deep neural network for binned time survival analysis; su-DeepBTS, semi-unsupervised deep neural network for binned time survival analysis.

In the external validation cohort, the performance of the su-DeepBTS algorithm was also the highest (C-index = 0.7077; AUC = 0.7224). When the model was trained using only 14 features selected as the optimal set for su-DeepBTS described in the feature selection part (next paragraph) and tested in the external validation cohort, it showed similar performance when all the features were used (C-index = 0.7013; AUC = 0.7123).

### Feature selection and performances of model-feature selector pairs

**Figure 1:**
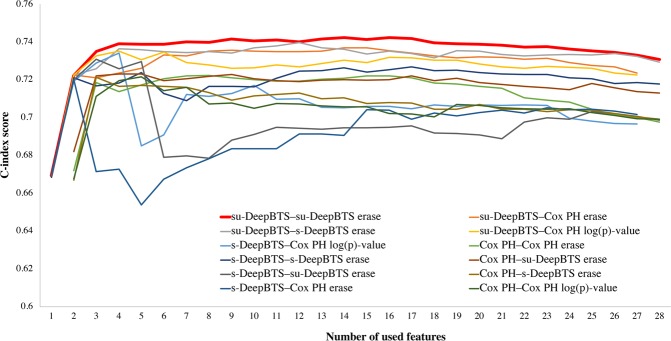
Comparison of model–feature selector pairs. The curves for all combinations of model–feature selector pairs are presented, with the *x*-axis representing the number of features used and the *y*-axis indicating the C-index.

Since three different models (Cox PH, s-DeepBTS, and su-DeepBTS) and four different selectors were employed, 12 pairs of models and feature selectors could be built in total (Table 3). The standard deviations of the ‘peak C-index’ and ‘Area under the graph’ were 0.008 and 0.591, respectively; therefore, the area under the graph was selected as a factor to determine the performances of the pairs. Interestingly, all four selectors with su-DeepBTS model showed good performances. The su-DeepBTS model using feature set ranked by su-DeepBTS selector exhibits best performance with the largest area under the graph and highest peak C-index score, as shown in Fig. 1. Therefore, the optimal model was defined as the su-DeepBTS model trained with top 14 feature sets selected by the su-DeepBTS selector.

**Table 3:** Performance comparison of model–feature selector pairs.

Pairs (model - feature selector)	Area under the graph	Peak score	Peak feature number
su-DeepBTS–su-DeepBTS erase	19.896134	0.742358	14
su-DeepBTS–s-DeepBTS erase	19.782187	0.739613	12
s-DeepBTS–s-DeepBTS erase	19.437697	0.726892	17
su-DeepBTS–Cox PH erase	18.982598	0.736879	14
su-DeepBTS–Cox PH log(p) value	18.912272	0.735058	4
s-DeepBTS–su-DeepBTS erase	18.835688	0.73088	3
Cox PH–su-DeepBTS erase	18.683417	0.723161	5
Cox PH–Cox PH erase	18.587178	0.72231	7
Cox PH–s-DeepBTS erase	18.41109	0.717018	5
s-DeepBTS–Cox PH log(p) value	18.375609	0.734164	4
Cox PH–Cox PH log(p) value	18.358491	0.72157	5
s-DeepBTS–Cox PH erase	17.984331	0.719938	2
Standard Deviation	0.591	0.008	—

Each row presents area under the graph drawn in Fig. 1. with the number of features used as the x-value and C-index as the y-value (“Area under the graph” column), peak C-index score in each graph (“Peak score” column), and the number of features used when the C-index score is maximum (“Peak feature number” column).

Cox PH, Cox proportional-hazards; s-DeepBTS, supervised deep neural network for binned time survival analysis; su-DeepBTS, semi-unsupervised deep neural network for binned time survival analysis.

Table 4 lists the top 15 important features selected by the four different feature selectors. In the case of su-DeepBTS erasing feature selector, gender; ECOG performance status; baseline lung diffusion capacity (DLCO, diffusion capacity of carbon monoxide); laboratory findings including white blood cell (WBC) count, lymphocyte fraction, albumin; the pathological findings including the number of lymph node (LN) metastasis, T stage, tumor histology, vascular invasion, and lymphatic invasion; achievement of complete resection (R0 resection); neoadjuvant treatment and adjuvant treatment were identified as the optimal 14 feature set for su-DeepBTS.

**Table 4:** Top 15 important features selected by four different feature selectors.

	Cox PH log(p) value (ascending order)	Cox PH erasing feature selection	s-DeepBTS erasing feature selection	su-DeepBTS erasing feature selection
1	No. of LN positivity	No. of LN positivity	No. of LN positivity	No. of LN positivity
2	T stage	T stage	T stage	T stage
3	ECOG	WBC	Age	R0 resection
4	Vascular invasion	Sex	R0 resection	Sex
5	WBC	Lymphocyte fraction	Vascular invasion	Vascular invasion
6	Adjuvant treatment	DLCO	WBC	DLCO
7	Age	CEA	Tumour differentiation	Lymphocyte fraction
8	CEA	Vascular invasion	Lymphatic invasion	WBC
9	CRP	Haemoglobin	Perineural invasion	ECOG
10	Tumour size	Tumour differentiation	DLCO	Lymphatic invasion
11	Lymphocyte fraction	Albumin	Tumour size	Histology
12	Tumour differentiation	ECOG	ECOG	Neoadjuvant treatment
13	DLCO	Smoking	LDH	Adjuvant treatment
14	Histology	Adjuvant treatment	Albumin	Albumin
15	Perineural invasion	Tumour size	Haemoglobin	Tumour differentiation

Cox PH, Cox proportional-hazards; s-DeepBTS, supervised deep neural network for binned time survival analysis; su-DeepBTS, semi-unsupervised deep neural network for binned time survival analysis; LN, lymph node; ECOG, Eastern Cooperative Oncology Group; WBC, white blood cell; DLCO, diffusion capacity of carbon monoxide; CEA, carcinoembryonic antigen; LDH, lactic acid dehdrogenase

### External validation

To confirm that the proposed model would be effective when applied to a completely different dataset, the trained model was tested using an external validation cohort. Since the optimal feature set for each model had been determined, the test scores of the external validation cohort for the whole feature set and the set of the optimal features for each model were obtained. As summarized in Table 2, the su-DeepBTS model also outperformed the other models when applied to the external validation dataset in the entire as well as optimal feature set case. All of the test scores obtained using the external validation set are shown in Supplementary Table S2 (all features) and Supplementary Table S3 (optimal features for each model).

Additional experiments were also conducted with the public dataset (Supplementary Tables S4 and S5). In the common set, su-DeepBTS outperformed the Cox PH model, which suggested that the former could be scalable to predict the survival of different cancer types.

### Prediction of 3-year recurrence risk

**Figure 2:**
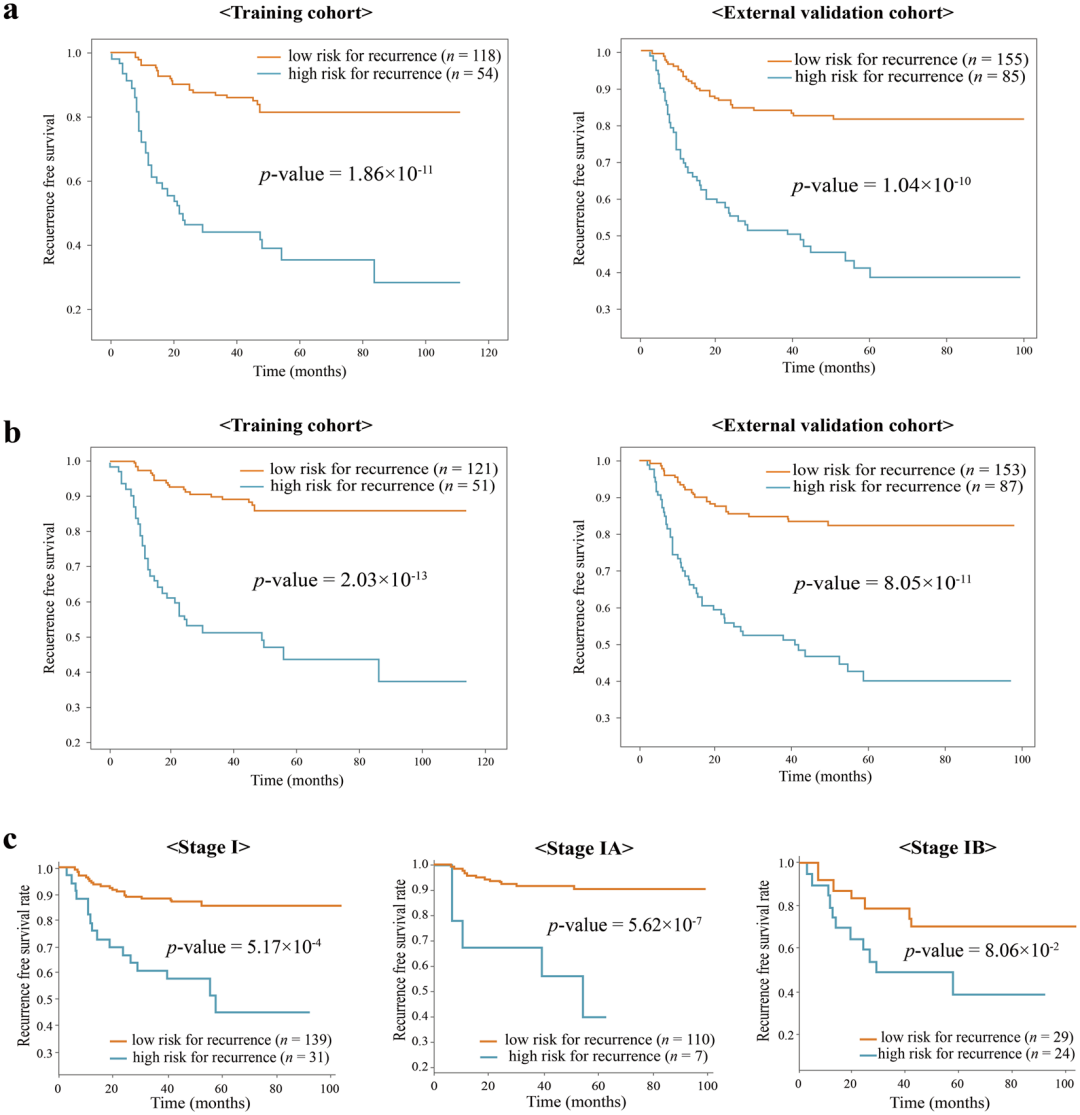
Kaplan–Meier curves according to the predicted risk of recurrence for all patients which obtained using su-DeepBTS model trained with (**a**) optimal 14 features and (**b**) all 28 features (left side is for the training cohort and right side is for the external validation cohort). (**c**) Kaplan–Meier curves according to predicted risk of recurrence in stage I/IA/IB patients of external validation cohort which obtained using su-DeepBTS model trained with optimal 14 features.

To evaluate the efficacy of the su-DeepBTS model trained using the top 14 features selected by the su-DeepBTS selector, the confusion matrix was obtained by comparing the predicted high- and low-risk groups for the 3-year recurrence. To divide the predicted high- and low-risk groups, the threshold value was defined as the value at the point farthest from the $y=x$ line in the receiver operating characteristic (ROC) curve obtained after calculating the AUC. The sensitivity, specificity, and accuracy were calculated based on the true label for the 3-year recurrence (Table 5). Because there were two different cohorts (the training and external validation cohorts) and two different feature sets (the whole feature set and optimal feature set), four different cohort–feature set pairs could be used to evaluate the su-DeepBTS model. The Kaplan–Meier curves of the external validation cohort show similar performances to those of the training cohort, demonstrating the significant difference in survival prognoses between the predicted high- and low-risk groups (Fig. 2). Notably, in both the cohorts, the *p*-value of the results obtained using only 14 features are almost the same as those obtained using all of the features.

**Table 5:** Sensitivity, specificity, and accuracy for 3-year recurrence prediction using su-DeepBTS model.

Training cohort	Sensitivity	Specificity	Accuracy
number of features = 14	0.72047143	0.73982096	0.73432601
number of features = 28	0.68479412	0.76053801	0.73782872
**Validation cohort**	**Sensitivity**	**Specificity**	**Accuracy**
number of features = 14	0.621875	0.74204545	0.7100
number of features = 28	0.634375	0.73522727	0.7083333

In addition, the analysis was performed for stage I patients only, which is of special interest owing to the controversies among clinicians regarding whether postoperative treatment should be performed for such patients. The recurrence-free survival (RFS) durations of patients with high and low risk scores were significantly different (lowest *p*-value = 3.99 × 10^−7^, average *p*-value = 5.17 × 10^−4^, Fig. 2c < Stage I>), even in stage IA (lowest *p*-value = 5.62 × 10^−7^, average *p*-value = 1.85 × 10^−1^, Fig. 2c < Stage IA>) and stage IB (lowest *p*-value = 8.05 × 10^−2^, average *p*-value = 1.69 × 10^−1^, Fig. 2c < Stage IB>).

## Discussion

In the clinical Big Data era, an approach using neural network can serve as alternatives to the Cox PH model that overcome the disadvantages of the latter. In this study, we developed a deep learning algorithm using a negative log likelihood (NLLH) cost function to predict the clinical outcomes in particular time intervals of NSCLC patients who received surgical resection by using clinico- pathological data, which is easily achievable in actual clinical practice. The su-DeepBTS model yielded the best performance among the three models employed to predict cancer recurrence and also performed well as a feature selector. Since prognostic analysis and feature selection can be conducted simultaneously using the su-DeepBTS model, it could serve as an important means of applying deep learning to predict recurrence and extract major features from electronic hospital record data.

Our proposed deep learning model is more useful for survival analysis than the traditional statistical method, the Cox PH method. First, the assumption of proportional hazards is not needed. Second, the neural network training models can handle all of the samples, including censored samples and tied samples, without any additional approximations for survival analysis. Third, the su-DeepBTS model can capture the non-linearity of the features because it is a multi-layer perceptron (MLP) model. Moreover, in comparison with the current discrete-time survival analysis based on neural network, the bias caused by manual generation of output vector can be reduced because su-DeepBTS is an unsupervised model and therefore does not need specific outputs.

Previous survival analysis studies using machine learning involved the use of support vector machine-based prediction models (sensitivity = 0.89; specificity = 0.73) or decision support systems (C-index = 0.84 with an accuracy of 86%) to predict breast cancer recurrence^9,10^, and probabilistic neural networks to predict cervical cancer recurrence (sensitivity = 0.975; accuracy = 0.892)^11^. In their research on lung cancer, Lynch *et al*. compared various supervised machine learning classification techniques using the Surveillance, Epidemiology, and End Results (SEER) database and showed that the models in which the gradient boosting machine was utilized with the root-mean-squared error (RMSE) were the most accurate^12^. However, these models are not suitable for identifying patients with high and low risks of recurrence at particular time point. The su-DeepBTS can overcome this problem because it is a multi-task learning model that can calculate the survival probability of each time-bin, incorporating prediction tasks in multiple time intervals into a single learning process^13^.

Interestingly, in terms of feature selection, the su-DeepBTS model was also the most effective. The su-DeepBTS model achieved the highest performance with the optimal feature set selected by the su-DeepBTS selector, and the same was also observed with the external validation cohort. Because adding other features to the optimal feature set degrades model performance, creating a relevant feature set is crucial for survival analysis, and the set can be optimized by the su-DeepBTS selector. Thus, the su-DeepBTS model is better suited for handling a huge amount of clinical data, not only as a recurrence predictor, but also as a feature selector.

The top 14 features selected from the su-DeepBTS included well-known prognostic parameters that are clinically relevant. Notably, the number of metastatic LNs was selected as a common top feature by four feature selectors and this number is associated with prognosis in various malignancies, including colorectal, gastric, breast, and bladder cancer^14^. On the other hand, in lung cancer, the N stage is determined by the locations of metastatic LNs other than the number. However, in our study, the number of metastatic LNs outweighed the significance of the N stage as the prognostic feature. Thus, the prognostic significance of this parameter could be validated in future studies.

Stage I patients are of special interest within this population as substantial intervention is a matter of debate. To determine whether the su-DeepBTS model could differentiate among the prognoses of early-stage NSCLC patients, the survival probability according to the risk identified by the su- DeepBTS model was calculated for the stage I patients. The RFS durations of patients with high and low risk scores were significantly different from one another, even when the analysis was performed separately for stages IA and IB. These results imply that the model can further sub-categorize stage I patients who are at risk of recurrence and might need substantial treatment. Thus, using the su-DeepBTS model trained with the selected optimal features could be an effective method of survival analysis.

Our study has several limitations. First, about 70% of the samples were censored in the training and external validation cohorts, which could have negatively impacted the model performance. Nevertheless, we were able to create a deep learning-based survival analysis model with better performance than the Cox PH model by using an NLLH loss function. As a future step, we will increase the number of samples, especially the number of relapsed patients, to obtain a comparable ratio between relapsed and censored patients. In addition, some data used for model training and validation were missing or inaccurate due to the retrospective nature of the study. As computational prognostic prediction is highly dependent on the data quality, the clinical utility of our proposed model remains to be established prospectively.

In conclusion, we developed a novel semi-unsupervised binned-time survival analysis algorithm using clinico-pathological parameters. The su-DeepBTS model using 14 features selected by the su- DeepBTS selector could predict the prognoses of resected NSCLC patients better than the Cox PH model. Since prognostic analysis and feature selection can be performed simultaneously with this algorithm, it provides a useful means of applying deep learning to extract major features from electronic hospital record data and performing analysis for clinical informatics. In addition, as multi-modal data integration is important for accurate prognosis prediction, we plan to merge various features in radiological and pathological imaging, and genomic data into input features of this model for performance enhancement.

## Methods

### Study population

Two cohorts of NSCLC patients who received surgical resection were enrolled in this study. The inclusion criteria were patients with NSCLC histology and having received surgical resection with a curative aim. Patients with minimally invasive adenocarcinoma, adenocarcinoma *in situ*, or bronchoalveolar carcinoma, were excluded. The training cohort consisted of 1,022 patients who were treated between January 2010 and March 2015. The external validation cohort of 298 patients was obtained between April 2015 and December 2016. Clinical and pathological data with 30 variables were retrospectively collected by eight independent reviewers. The age, gender, smoking history, ECOG performance status, laboratory findings, and pulmonary function of each patient were obtained within 2 weeks before the date of surgery. The tumor size was defined as the longest sample diameter. The TNM stage was pathologically classified according to the 7^th^ edition of the American Joint Committee on Cancer^15^. Neoadjuvant or adjuvant chemotherapy consisted of platinum-based doublet agents. Follow-up computed tomography of the chest was performed for each patient in 3–4 month intervals for the first 2 years after surgical resection, and every 6 months thereafter. This study was approved by the institutional review board of Catholic Medical Center (No.UC17SESI0073) and was performed in accordance with the guidelines of human research. The requirement for written informed consent was waived by the institutional review board (Catholic Medical Center) because of the study analysis being retrospective in nature.

### Data preparation and processing

Before building the models, missing values of the categorical features were filled with 10,000 and those of the continuous features were filled with the averages of the existing values. The Cox PH model was used as the baseline model, and therefore, it was necessary to exclude the variables that violated the PH assumption to avoid building an ill-fitted Cox PH model. Among the five variables that violated the assumption, ‘creatinine’ and ‘smoking amount’ features were excluded from the input features owing to their low importance in the fitting of the Cox PH model. In addition, the ‘R0_resection’ feature was used as stratifying factors because it is important for Cox PH fitting^16^. A detailed explanation of dataset processing is presented in the Supplementary methods. Consequently, 28 out of 30 features were used to train the models.

To test all of the samples in the processed training cohort, five-fold cross-validation was performed, which means that the whole dataset was divided into five sets, among which one was employed as a test set and the other four were used to learn the model, and the test score was obtained from the test set. When dividing the dataset, the percentage of censored patients in the entire sample was set to be the same in the training and test sets. The final test score was defined as the test score averaged over 10 iterations of five-fold cross-validation to consider the change of the score depending on which sample was included in each training and test set. This scoring method was equivalently applied to the external validation cohort, except that external validation cohort was used to obtain the test score of the trained model in each fold.

### Binned-time survival analysis models

#### Supervised binned-time survival analysis (s-DeepBTS)

The s-DeepBTS model is a supervised single-layer perceptron model using the RMSE as the loss function. To train the model, the proper output, i.e., the survival probability in each time interval, must be pre-defined. For relapsed patients, *y*_*j*_, the output value of the *j*^th^ time interval *I*_*j*_, is 1 when the patient is alive without recurrence, and 0 after the patient shows recurrence. For censored patients, *y*_*j*_ is 1 until the follow-up is lost, and $\prod _{i={t}_{i}\le {I}_{j}}(\frac{1-{d}_{i}}{{n}_{i}})$ after censoring occurs, where *n*_*i*_ is the total number of samples without recurrence at the beginning of *I*_*j*_ and *d*_*i*_is the number of event-occurred samples in the specific *I*_*j*_. The total number of time intervals *J* is defined as

1 $$\forall j\in [[1,\,J]],\,{I}_{j}=[{t}_{j-1},\,{t}_{j}),\,{\rm{with}}\,{t}_{0}=0\,{\rm{and}}\,{t}_{J}=int(\max (RFS))+1.$$

#### Semi-unsupervised binned-time survival analysis (su-DeepBTS)

The su-DeepBTS model is a semi-unsupervised MLP model that can capture the non-linearity of the input features. To predict the hazard probability in each time interval in an unsupervised mode, the custom loss was constructed to calculate the NLLH functions of the patients in each time interval and add them up, and the result was used as the final loss value of model. The model was trained to minimize loss. The loss function is

2 $$NLLH(\beta )=-{\sum }^{}\,log\left(\frac{{e}^{\beta {X}_{j}}}{{\sum }_{I\in {R}_{j}}{e}^{\beta {X}_{j}}}\right).$$

In Eq. (2), *β* is the regression coefficient, *R*_*j*_ is an at-risk sample for which an event may occur at time *j*, *X*_*j*_ is the value of the explanatory variable for the individual for which the event occurred at time *j*, and $\sum _{I\in {R}_{j}}{e}^{\beta {X}_{j}}$ is the sum of the risks for members of the at-risk set *R* at time *j*.

**Figure 3:**
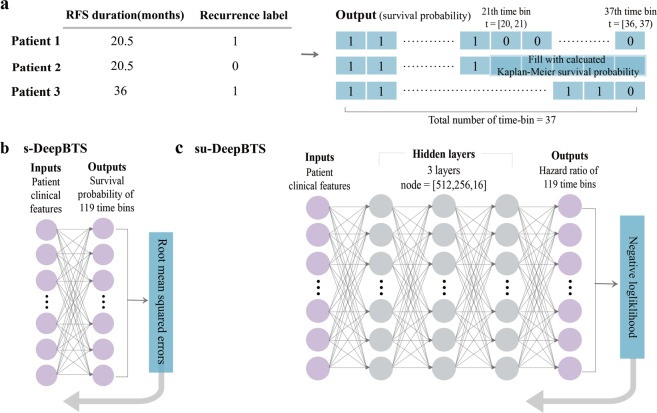
Overview of the proposed binned-time survival analysis models. (**a**) Simple example to explain the method of calculating survival probabilities for building output values. The total time-bin count of the output is based on the maximum RFS duration among all of the samples. Since 36 months is the longest duration defined, the total number of bins is 37. Each bin was filled with a survival probability value according to the recurrence statuses of the samples. For all of the samples, each time bin was filled with 1 until recurrence or follow-up loss. After relapse or follow-up loss, the time bin was filled with 0 for recurrence patients and with the calculated Kaplan–Meier survival probability for censored patients. Schema of of (**b**) s-DeepBTS and (**c**) su-DeepBTS models. RFS, recurrence-free survival; s-DeepBTS, supervised deep neural network for binned time survival analysis; su-DeepBTS, semi-unsupervised deep neural network for binned time survival analysis.

The main concept of both models is multi-task learning, as in the existing models^7^, but the means of obtaining the output is simplified in the s-DeepBTS model and no pre-defined output is required to train the su-DeepBTS model. Overviews of the complete processes of these models are provided in Fig. 3. As the baseline of the two proposed models, the traditional statistical survival analysis model, the Cox PH model, was used. Each model is described in detail in the Supplementary Methods.

### Feature selection

Determining how each feature affects the model performance is an important step for further application. Deep-learning models do not generate specific feature importance indicators, so a novel feature selection method had to be created to extract the importance of each feature from the proposed models. The newly created feature selection method called “erasing feature selection” is a means of evaluating the model performance by excluding one feature at a time. A simple example is provided in Supplementary Fig. S1.

As the method of erasing feature selection can be applied to any model, all the three models proposed in this study can be used as feature selectors. Therefore, three selectors based on the erasing selection method (Cox PH/s-DeepBTS/su-DeepBTS erasing feature selection) and an additional selector based on log(p)-value extracted from Cox PH model (Cox PH log(p) value) were employed for feature selection.

### Statistical analysis

The RFS duration was defined as the time from the date of surgery until the first recurrence or death due to any cause, whichever was observed first, and the survival curves were estimated by utilizing the Kaplan–Meier method and compared using the log-rank test.

The performances of our models were measured and compared by employing two statistics, the C-index and AUC score. The weighted average was calculated using the estimated hazard probability in each time interval as a weight, and this calculated weighted average was set as the RFS duration, which was used as the input of the C-index. To determine the AUC of the classification for 3-year recurrence, patients censored before 3 years were excluded from the test set, because the recurrence labelling for those samples was not clear. After refining the test samples, the sigmoid function $(\frac{1}{1+{e}^{-x}})$ was applied to calculate the probability of 3-year recurrence. The value obtained by subtracting the predicted duration from the reference number of months (36 months) was used as the input of the sigmoid function, and the final result of the sigmoid function was used to calculate the AUC. These scoring methods were applied equivalently to the proposed models. In the Cox PH case, the RFS duration was predicted directly as an outcome of the model, so post-processing was not needed. The statistical analysis was performed using the ROC function in the *sklearn* package for the AUC and a custom function for C-index scoring. We applied the same analysis method for stage I subpopulation. A *p*-value less than 0.05 was considered statistically significant.

## Supplementary information

Supplementary data.

## Data Availability

The datasets generated during and/or analyzed during the current study are available from the corresponding author on reasonable request.
