# Supplementary material for: DeepBTS: Prediction of Recurrence-free Survival of Non-small Cell Lung Cancer Using a Time-binned Deep Neural Network
Source: Sci Rep. 2020 Feb 6;10:1952. doi: 10.1038/s41598-020-58722-z (PMC7005286; doi:10.1038/s41598-020-58722-z)
Supplement: Supplementary file 1 — Supplementary data. [file 41598_2020_58722_MOESM1_ESM.pdf]

DeepBTS: Prediction of Recurrence-free Survival of Non-small Cell Lung Cancer Using a Time-binned Deep Neural Network

Bora Lee[1], Sang Hoon Chun[2], Ji Hyung Hong[2], In Sook Woo[2], Seoree Kim[2], Joon Won Jeong[2], Jae Jun Kim[3], Hyun Woo Lee[4], Sae Jung Na[5], Kyongmin Sarah Beck[5], Bomi Gil[5], Sungsoo Park[1], Ho Jung An[2*], Yoon Ho Ko[2,6 *]

Data processing for Cox proportional hazard (PH) model

In a deep learning model, the input dataset involves few assumptions that must be satisfied for effective model training. However, in the case of a Cox PH model, there are certain mandatory assumptions that must be met. The most important assumption is that all individuals must have the same hazard function, but a unique scaling factor.

$$h_i(t) = b_i h(t) \tag{S1}$$

Because a scaling factor does not vary with time, the hazard ratio, which is the ratio of a hazard function of one individual to that of another individual is constant for all *t*

$$\frac{h_i(t)}{h_j(t)} = \frac{b_i h(t)}{b_j h(t)} = \frac{b_i}{b_j} \tag{S2}$$

As the above mentioned assumption cannot often be satisfied using a real-world dataset, it is necessary to verify whether the input variables can meet the mentioned assumption, that is, if the ratio of hazard function changes over time. Notably, the violation of the assumptions can lead to the creation of a false model. In "*lifelines*" package for survival analysis, the method that computes statistics to check the proportional hazard (PH) assumption is provided. There were five features, including R0-resction, Adjuvant-category, smoking-packyr, age-operation and Cr, of our training cohort that violated the assumption. For the training cohort to satisfy the PH assumption, we removed Cr (p-value = 0.9103) and smoking-packyr (p-value = 0.5701), which have relatively low importance in the fitting of the Cox PH model using the entire training cohort, and subsequently used R0-resection as a stratifying variable. This variable was used for subgrouping the entire sample according to the baseline hazards, and the hazard function was calculated for each divided group as follows.

$$h_{i|i \in Group}(t) = b_i h_{Group}(t) \qquad \text{(S3)}$$

Consequently, the training cohort satisfied the PH assumption, and the final feature sets for training both deep-learning and Cox PH models contained 28 out of the initial 30 features.

Deep learning models based on multi-task learning

Supervised binned-time survival analysis (s-DeepBTS)

To build the s-DeepBTS model, it was necessary to build the proper output first. For this purpose, the maximum recurrence free survival (RFS) duration (i.e., *max* (RFS)) in months was obtained and the time axis was divided into *J* time intervals. Here we set 1 month as the time interval, such that

$$\forall j \in [[1, J]], \quad I_j = [\ t_{j-1}, t_j) \text{ with } t_0 = 0 \text{ and } t_J = int(\max(RFS)) + 1.$$

(S4)

The response variable of each time interval y_j, which refers to the survival probability at a specific time point, was set differently according to the follow-up status. For the patients exhibiting recurrence, the survival probability y_j was 1 until the *k*-th bin satisfying $T_{event} \in I_k$ and 0 after the *k*-th bin. For the censored patients who did not show recurrence within the observation period or who were lost to follow-up, the calculated Kaplan–Meier survival probability for each time interval using all of the samples in our dataset was applied to y_j from the *k*-th bin satisfying $T_{censored} \in I_k$, and y_j was set to 1 until the *k*-th bin.

In summary, y_j = 1 until the patient exhibited recurrence or follow-up loss. After

that, y_j was simply set to 0 for the relapsed patient. In the case of the censored patient, y_j was calculated using the following equation.

$$y_j = \prod_{i=\ t_i\ \leq\ I_j} \left(\frac{1-d_i}{n_i}\right) \tag{S5}$$

where n_i is the total number of samples alive without recurrence at the beginning of I_j, d_i is the number of event-occurred samples in I_j, and equation (S5) is the Kaplan–Meier survival probability function corresponding to time interval j. This result set refers to the survival probability of each time interval. A simple example of the output-building method is provided in Fig. 3.

After obtaining these outputs for every subject, we built a multi-task regression model with a single-layer perceptron. To calculate the loss in each interval, we built a custom-loss function that calculated the root-mean of the summation of the squared errors between the true y_j and predicted y_j in each time interval. We used *RMSprop* function as the optimizer and a sigmoid function as the activation function which were defined in *Keras*.

Semi-unsupervised binned-time survival analysis (su-DeepBTS)

The supervised model is superior to the Cox PH model in that the former does not have to satisfy the proportional hazard assumption and can calculate the hazard function in a simple way considering tied events. However, since the created output values are based on the Kaplan–Meier survival function, the model exhibits better performance when there is no hidden layer. Thus, it is still powered by linear regression and fails to capture non-linearity from the data. To make a non-linear model, we developed a semi-unsupervised model with a

multi-layer perceptron. The number of output nodes and method of dividing the time axis are the same as those in the s-DeepBTS, but no specific probability value is assigned. To predict the hazard probability in each time interval in unsupervised mode, the custom loss was constructed to calculate the negative log-likelihood (NLLH) function of each patient in each time interval and to add them up, and the result was used as the final loss value of the model. The model was trained to minimize loss.

To construct the loss function, the likelihood function of the Cox PH model was used, and the formulae are:

Likelihood function : $L(\beta) = \prod_{j=1}^{J}\left(\frac{e^{\beta X_j}}{\sum_{I\in R_j} e^{\beta X_j}}\right)$

(S6)

Negative log likelihood function : $NLLH(\beta) = -\sum log\left(\frac{e^{\beta X_j}}{\sum_{I\in R_j} e^{\beta X_j}}\right).$ (S7)

In these formulae, β is the regression coefficient, R_j is an at-risk sample for which an event may occur at time j, X_j is the value of the explanatory variable for the individual at which the event occurred at time j, and $\sum_{I\in R_j} e^{\beta X_j}$ is the sum of the risks for members of the at-risk set R at time j. Because each output node of our model refers to the risk probability at that time point, the predicted value at each node corresponds to the $e^{\beta X_j}$ value of the above formulae. Thus, predicted output values were used as inputs of custom loss. This model was trained without an exact answer set, but it is not completely unsupervised because a binary table containing information about the at-risk set in each time interval must be used to calculate the loss function. Consequently, it can be called a semi-unsupervised model. It

consists of three hidden layers with batch-normalization and drop-out layers (dropout rate = 0.3). We used an *RMSprop* function as the optimizer and a sigmoid function as the activation function of the output layer which were defined in *Keras*.

Traditional model based on proportional hazard assumption: Cox PH model

Two novel models were compared to a statistical model, the Cox PH model, using the Python package "*lifelines*." The dataset was fitted to Cox PH model using the package class "*CoxPHFitter*." Then, using the fitted model, the RFS duration was predicted by employing the package function "*predict_expectation*."

External validation with the public survival related datasets

To verify the scalability of su-DeepBTS, which demonstrated the best performance in training/external validation cohort, additional experiments were conducted with public survival related datasets. Among the three datasets, two datasets, German Breast Cancer Study Group[1] and Molecular Taxonomy of Breast Cancer International Consortium[2] were obtained from the DeepSurv github repository[3], and NCCTG Lung Cancer Data[5] were available on the Rdatasets github repository[6]. The su-DeepBTS and Cox PH model were compared using the C-index score averaged using five iterations of five-fold cross validation. As shown in Supplementary Table 4, these datasets have different characteristics in terms of feature number, sample number, censored percentage and tied percentage. Although the number of features of public datasets is relatively lower than our own cohorts, as shown in Supplementary Table 5, su-DeepBTS outperforms the Cox PH model on all the public sets. Therefore, it can be inferred that su-DeepBTS can be applied to various datasets in real-world

scenarios, thereby producing valuable results.

Supplementary Tables

Supplementary Table S1. Detailed results of 10 iterations of five-fold cross-validation with the training cohort divided into five sets maintaining the proportion of event patients using all the 28 features.

Training Cohort	Cox PH		s-DeepBTS		su-DeepBTS	
	C-index	AUC	C-index	AUC	C-index	AUC
Iteration 1	0.70233794	0.73697702	0.69708449	0.72568702	**0.73296856**	**0.76788759**
Iteration 2	0.70567824	0.74077968	0.7167267	0.74333838	**0.73533431**	**0.77317093**
Iteration 3	0.70698534	0.74445133	0.70457967	0.73292558	**0.7272738**	**0.76438221**
Iteration 4	0.70796261	0.74348249	0.72511592	0.75962663	**0.73368587**	**0.7697822**
Iteration 5	0.70425728	0.73714115	0.71979371	0.75048966	**0.72359472**	**0.762238**
Iteration 6	0.68918003	0.72378618	0.71851864	0.75458898	**0.73108483**	**0.76973902**
Iteration 7	0.70242225	0.73336593	0.70835954	0.74058681	**0.73668229**	**0.77548699**
Iteration 8	0.70545462	0.73694873	0.7205733	0.74490058	**0.72980838**	**0.7671892**
Iteration 9	0.70840902	0.74528548	0.70544412	0.73049621	**0.72597923**	**0.75869512**
Iteration 10	0.71578604	0.74824378	0.70987139	0.7373449	**0.72938872**	**0.7682476**
Average	0.70484734	0.73904618	0.71260675	0.74199848	**0.73058007**	**0.76768189**

Cox PH, Cox proportional-hazards; AUC, area under the curve; s-DeepBTS, supervised deep neural network for binned time survival analysis; su-DeepBTS, semi-unsupervised deep neural network for binned time survival analysis

Supplementary Table S2. Detailed results of 10 iterations of model training with five-fold cross-validation with the training cohort divided into five sets maintaining same event patient ratio and testing with an external validation cohort using all the 28 features.

Number of features = 28	Cox PH		s-DeepBTS		su-DeepBTS	
	C-index	AUC	C-index	AUC	C-index	AUC
Iteration 1	0.69419182	0.70770597	0.69113303	0.70298295	**0.70740762**	**0.72215909**
Iteration 2	0.69291927	0.70509588	0.69183352	0.70775923	**0.70588991**	**0.7207919**
Iteration 3	0.69669021	0.70933949	0.6835211	0.69405185	**0.70801471**	**0.72313565**
Iteration 4	0.69443699	0.70704901	0.6915183	0.70494496	**0.70731423**	**0.72208807**
Iteration 5	0.69643337	0.70857599	0.67679645	0.69176136	**0.70494425**	**0.71898082**
Iteration 6	0.69370148	0.70585938	0.68778238	0.70319602	**0.70915883**	**0.72402344**
Iteration 7	0.69309439	0.70498935	0.68931177	0.70514915	**0.70633355**	**0.72134233**
Iteration 8	0.69444866	0.70605469	0.68557586	0.69881037	**0.71161053**	**0.72693537**
Iteration 9	0.69117973	0.705087	0.69116806	0.70601918	**0.70722083**	**0.72212358**
Iteration 10	0.69184519	0.70454545	0.6902691	0.70575284	**0.70883194**	**0.72286932**
Average	0.69389411	0.70643022	0.68789096	0.70204279	**0.70767264**	**0.72244496**

Cox PH, Cox proportional-hazards; AUC, area under the curve; s-DeepBTS, supervised deep neural network for binned time survival analysis; su-DeepBTS, semi-unsupervised deep neural network for binned time survival analysis

Supplementary Table S3. Detailed results of 10 iterations of model training with five-fold cross-validation with the training cohort divided into five sets maintaining same event patient ratio and testing with an external validation cohort using the optimal features for each model.

Optimal feature set	Cox PH		s-DeepBTS		su-DeepBTS	
	C-index	AUC	C-index	AUC	C-index	AUC
Iteration 1	0.69317028	0.71188743	0.69372483	0.70697798	**0.700507851**	**0.71091974**
Iteration 2	0.69150079	0.71036932	0.69389995	0.70834517	**0.697974432**	**0.70843395**
Iteration 3	0.69094624	0.70983665	0.69511412	0.70882457	**0.70079972**	**0.71221591**
Iteration 4	0.69258654	0.71160334	0.6950324	0.70815874	**0.702399159**	**0.71212713**
Iteration 5	0.69381239	0.71277521	0.69404004	0.70779474	**0.703169692**	**0.71439986**
Iteration 6	0.6932053	0.71215376	0.69428521	0.70887784	**0.699959138**	**0.71173651**
Iteration 7	0.69165256	0.70988104	0.69495651	0.70965909	**0.701395132**	**0.71257102**
Iteration 8	0.69221295	0.71080433	0.69554609	0.71006747	**0.704360516**	**0.71596236**
Iteration 9	0.69186271	0.71057351	0.69259238	0.70578835	**0.699854066**	**0.71056463**
Iteration 10	0.69298348	0.71179865	0.69444866	0.70896662	**0.702083941**	**0.71361861**
Average	0.69239332	0.71116832	0.69436402	0.70834606	**0.701250365**	**0.71225497**

Cox PH, Cox proportional-hazards; AUC, area under the curve; s-DeepBTS, supervised deep neural network for binned time survival analysis; su-DeepBTS, semi-unsupervised deep neural network for binned time survival analysis

Supplementary Table S4. Description of public survival related datasets

Datasets	Num. features	Num. samples	Num. (%) censored	Num. (%) unique time
**LUNG**	7	228	63 (28)	186 (82)
**GBSG**	7	2232	965 (43)	1230 (55)
**METABRIC**	9	1904	801 (42)	1686 (89)

Lung, NCCTG Lung Cancer Data[5] available on the Rdatasets github repository[6] ; GBSG, German Breast Cancer Study Group[1] available on DeepSurv github repository[3]; METABRIC, Molecular Taxonomy of Breast Cancer International Consortium[2] available on DeepSurv github repository[3]

Supplementary Table S5. Comparison of model performance with public survival related datasets using the C-index

Dataset	Cox PH	su-DeepBTS
**LUNG**	0.594629±0.0140	**0.610010±0.0116**
**GBSG**	0.662576±0.0004	**0.677715±0.0014**
**METABRIC**	0.635081±0.0016	**0.644970±0.0136**

Lung, NCCTG Lung Cancer Data[5] available on the Rdatasets github repository[6] ; GBSG, German Breast Cancer Study Group[1] available on DeepSurv github repository[3]; METABRIC, Molecular Taxonomy of Breast Cancer International Consortium[2] available on DeepSurv github repository[3]

Supplementary Figures

Supplementary Figure 1. The erasing feature selection method was developed to provide insights into feature importance in deep-learning models. In short, this method measures the importance of each feature based on the prediction performance of the model trained without specific features. As an example, let us assume that there are three features in the input data, and two cycles are required to complete the feature importance set. In the first cycle, three different experiments are conducted by erasing three features, one by one. In the first cycle, the feature that maximizes the test score of each experiment, in this case, F3, is selected as the insignificant feature and ranked as the least important. The second cycle is performed with the feature set excluding feature F3. In this cycle, the average score of the current and previous cycle used for criteria of feature importance to reduce the error from the difference of entire feature set at each cycle. As shown, F1 was ranked the most important feature because model performance was worse when F1 was excluded. By repeating this process for all the features, the unimportant features are eliminated one by one until, causing only the most important feature to remain, and the feature important ranks were determined in the reverse order of deletion.

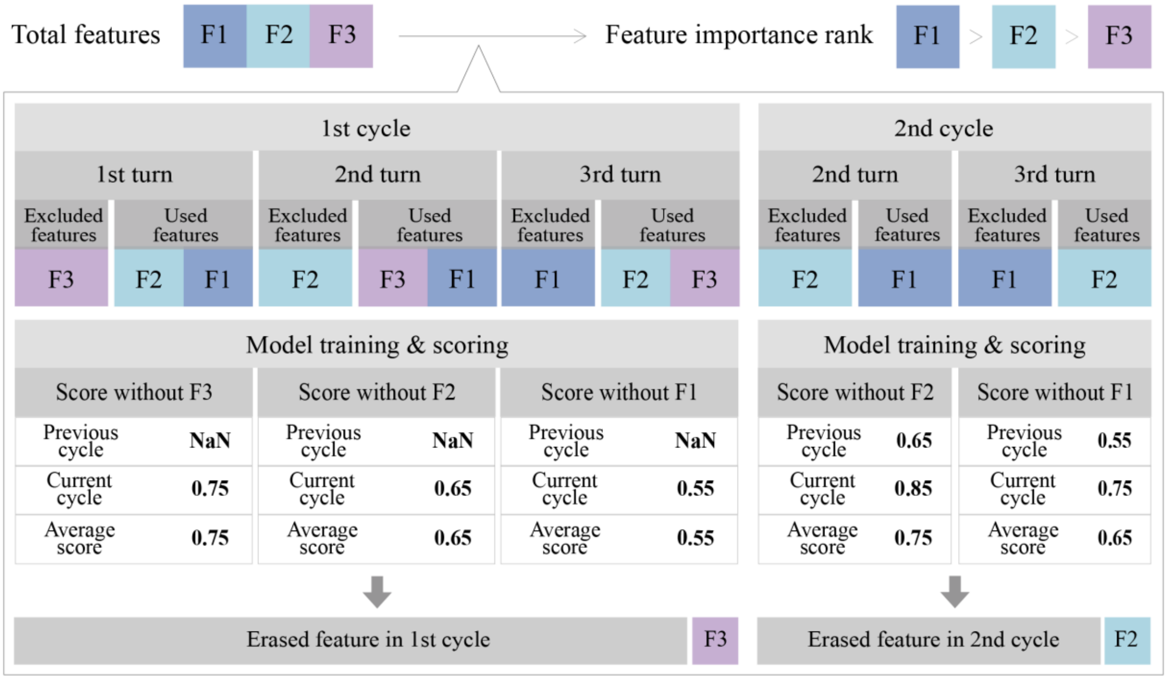

References

1 Schumacher, M. *et al.* Randomized 2 x 2 trial evaluating hormonal treatment and the duration of chemotherapy in node-positive breast cancer patients. German Breast Cancer Study Group. **12**, 2086-2093 (1994).

2 Curtis, C. *et al.* Langerød A. **2**, 346-352 (2012).

3 *https://github.com/jaredleekatzman/DeepSurv*, <https://github.com/jaredleekatzman/DeepSurv> (

4 Grambsch, T. T. a. P. *Modeling Survival Data: Extending the Cox Model*. (Springer-Verlag, New York, 2000).

5 Loprinzi, C. L. *et al.* Prospective evaluation of prognostic variables from patient-completed questionnaires. North Central Cancer Treatment Group. **12**, 601-607 (1994).

6 *https://vincentarelbundock.github.io/Rdatasets/datasets.html*, <https://vincentarelbundock.github.io/Rdatasets/datasets.html> (
